# Decrease of Pirimiphos-Methyl and Deltamethrin Residues in Stored Rice with Post-Harvest Treatment

**DOI:** 10.3390/ijerph110505372

**Published:** 2014-05-16

**Authors:** Chuanshan Yu, Yanjie Li, Qian Zhang, Nan Zou, Kejia Gu, Xuesheng Li, Canping Pan

**Affiliations:** 1 Department of Applied Chemistry, College of Science, China Agricultural University, 2 Yuanmingyuanxilu, Haidian District, Beijing 100193, China; E-Mails: chuanshan.yu@gmail.com (C.Y.); gqblyj@163.com (Y.L.); zhangqian12016@163.com (Q.Z.); zounan@cau.edu.cn (N.Z.); kjgu@cau.edu.cn (K.G.); 2 Institute of Pesticide and Environmental Toxicology, Guangxi University, 100 daxuedonglu, Nanning 530005, China; E-Mail: lxsnngx@163.com

**Keywords:** pirimiphos-methyl, deltamethrin, rice, post-harvest treatment, residue, dissipation

## Abstract

A modified quick, easy, cheap, effective, rugged (QuEChERS) method with multi-walled carbon nanotubes (MWCNTs) as reversed-dispersive solid phase extraction (r-DSPE) material was applied to the analysis of pirimiphos-methyl and deltamethrin residues in stored rice. Two dustable powder (DP) formulations (2% pirimiphos-methyl and deltamethrin DP; 5% pirimiphos-methyl DP) were applied in simulated storehouse trials in the lab. The residues and dissipation of the two pesticides in stored rice were investigated. Slow dissipation of both pesticides was observed in stored rice. The half-lives of pirimiphos-methyl were 23.9–28.9 days, and those of deltamethrin were 23.9–24.8 days. Residues of pirimiphos-methyl from application rates of 4.5–6.75 a.i. mg/kg (active ingredient milligram per kilogram) and 10–15 a.i. mg/kg were 1.6–3.8 mg/kg and 3.0–4.5 mg/kg at 60 days Pre-harvest Interval (PHI). Residues of deltamethrin from an application rate of 0.5–0.75 a.i. mg/kg were 0.13–0.14 mg/kg at 60 days PHI. Both pesticides residues were below the Maximum Residue Limits (MRLs) established by the Codex Alimentarius Commission (CAC). Therefore, at the recommended dosages they are safe for use on stored rice.

## 1. Introduction

Pesticides benefit grain storage in controlling the losses caused by insect pests. Some pesticides can be applied directly to the commodity and pesticide residues from these post-harvest treatments may be hazardous to human health [1]. The environment in the storehouse and the moisture content in stored grain will affect the dissipation of organophosphate and pyrethroid pesticides from grains [2], therefore the behaviors of residues of pesticides applied to stored grain should be investigated. 

**Figure 1:**
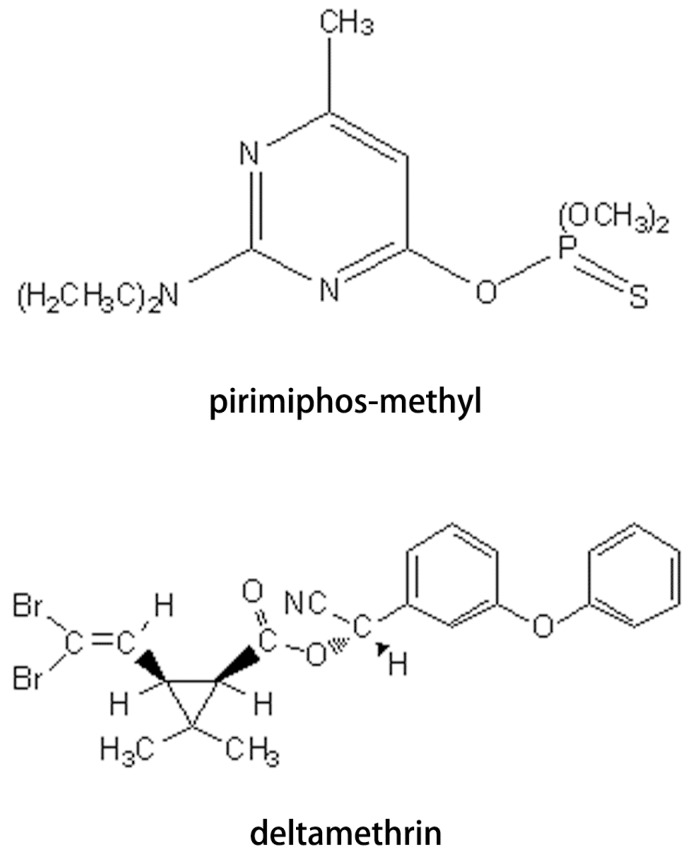
Chemical structures of pirimiphos-methyl and deltamethrin.

Rice is a main source of food and increasing rice productivity depends on large-scale application of pesticides, including pesticides used in storehouses [3]. Pirimiphos-methyl (Figure 1) is widely used in the major grain-producing countries to protect against insect attack, and this organophosphate insecticide has long insecticidal activity persistence [4]. Deltamethrin (Figure 1), a pyrethroid insecticide, is considered to be of relatively low toxicity compared to other insecticides [5]. It is also currently available for controlling insect pests in stored products [6]. Pirimiphos-methyl and deltamethrin are usually used in stored grains directly, making pesticide residues a problem. Maximum residue limits (MRLs) of pirimiphos-methyl and deltamethrin in rice have been set by the European Union (5 mg/kg and 2 mg/kg), USA (8 mg/kg and 1 mg/kg), China (0.5 mg/kg for deltamethrin) and the Codex Alimentarius Commission (CAC, 7 mg/kg and 2 mg/kg). 

In the analysis of pesticide residues, effective extraction and cleanup methods are necessary, such as liquid-liquid extraction [7,8], solid phase extraction [9,10,11] and dispersive solid phase extraction (dSPE) [12]. The rice matrix is rich in starch, which makes the pretreatment complex, especially the cleanup and filtering steps. Many extraction methods for analysis of pesticides residues in grain require different cleanup steps. The QuEChERS method by Anastassiades *et al.* [13] is widely used. QuEChERS does well in the extraction and cleanup, but it still need an appropriate absorbent material to complete the cleanup step, such as graphite carbon black (GCB), primary secondary amine (PSA) [14,15] or MWCNTs [16,17]. MWCNTs have special physical and chemical characteristics that can be exploited, such as unique thermal, mechanical, electronic and chemical properties. MWCNTs performed well in cleanup of tea, tobacco, cabbage, spinach, grape and orange matrices [16,17,18,19,20]. In this work, a modified QuEChERS method with MWCNTs as r-DSPE material coupled with GC-FPD and GC-ECD was applied to evaluate the dissipation dynamics and residues of pirimiphos-methyl and deltamethrin in stored rice. The residue data collected from storage trials in rice should be helpful to minimize the risks to human health.

## 2. Experimental Section

### 2.1. Chemicals and Reagents

Pirimiphos-methyl (98.0%) and deltamethrin (99.5%) were obtained from the Institute of the Control of Agrochemicals, Ministry of Agriculture, People’s Republic of China. Commercial 2% pirimiphos-methyl (1.8%) and deltamethrin (0.2%) dustable powder (DP) and 5 % pirimiphos-methyl DP were purchased from Hunan Haili Chemical Industry Co., Ltd. (Hunan, China). HPLC-grade acetonitrile was obtained from Fisher Chemicals (Fair Lawn, NJ, USA). Analytical grade anhydrous sodium chloride (NaCl) and magnesium sulfate (MgSO_4_) were obtained from sinopharm chemical Reagent (Beijing, China). MWCNTs with average external diameters of 10–20 nm, 5 nm i.d. (inner diameter) were provided by Bonna-Agela Technologies (Tianjin, China). MWCNTs were dried for 2 h at 120 °C to remove the absorbed water and then kept in desiccators for storage. Ultra-pure water was obtained from a Milli-Q system (Millipore, Bedford, MA, USA).

### 2.2. Trials

The storage trials, including the dissipation and residue experiments were carried out in Beijing and Jiangsu in 2011. The valve bags were 70 cm (length) × 50 cm (width). They were used to isolate different plots. Altogether, 27 valve bags were prepared, each containing 2 kg of rice (excluding husked, including polished). One was used as control treatment with no applied pesticide. One was used for 2% pirimiphos-methyl and deltamethrin DP residue dynamics experiment and 12 for the final residue tests. One was used for 5 % pirimiphos-methyl DP residue dynamics experiment and the last 12 for the final residues tests.

In the dissipation dynamic tests, 2% DP and 5% DP were applied at 7.5 a.i. mg/kg and 15 a.i. mg/kg, respectively, by mixing with the stored rice. Both dosages were 1.5 times the recommended dosage of the two formulations. Representative samples were collected at 0 (2 h after treatment), 3, 7, 14, 21, 30, 45 and 60 days after application. The rice samples were stored at −20 °C for further analysis.

The final residue tests were designed to apply two dosages for each formulation: a low-level dosage (recommended dosage of 5 a.i. mg/kg of 2% DP and 10 a.i. mg/kg of 5% DP) and a high-level dosage (7.5 a.i. mg/kg of 2% DP and 15 a.i. mg/kg of 5% DP). These four treatments were conducted separately in triplicate. Representative samples were collected from each bag at Pre-Harvest Intervals (PHIs) of 30 and 60 days for 2% DP, 45 and 60 days for 5% DP after the final application. The rice samples were stored at −20 °C for further analysis.

### 2.3. Analytical Procedures

A thoroughly homogenized sample (10 g) was weighed into a 50 mL Teflon centrifuge tube. Water (3 mL) and acetonitrile (10 mL) were added and the tube was shaken vigorously for 1 min with a vortex mixer ensuring that the solvent interacted well with the entire sample. Anhydrous NaCl (1 g) and anhydrous MgSO_4_ (4 g) were added into the mixture, followed again by 1 min vortexing. After centrifugation (3,800 rpm, 5 min), 1 mL of the clarified supernatant was introduced into a 2 mL micro-centrifuge tube containing MWCNTs (10 mg) and MgSO4 (150 mg). Then the mixture was shaken vigorously for 1 min and centrifuged for 1 min at 10,000 rpm with a microcentrifuge. Finally the acetonitrile layer was filtered through a 0.22 µm filter membrane and the extract was placed in a GC vial to carry out the chromatographic analysis.

### 2.4. Instruments and Chromatographic Conditions

An Agilent 6890N Network GC system (Agilent Technologies, Palo Alto, CA, USA) equipped with a 7683B Series splitless auto-injector, a 7683B Series auto-sampler and an Electron Capture Detector (ECD) was used for analysis of deltamethrin. An Agilent Technologies Capillary Column HP-5 analytical column (30 m × 0.25 mm i.d. × 0.25 µm film thickness) was used for the GC separations, with nitrogen as carrier gas at a constant flow rate of 1.0 mL/min. The column temperature was initially at 190 °C (hold for 1 min), increased to 270 °C at the rate of 30 °C/min and kept there for 16.3 min. The temperatures of the injector port and detector were 260 °C and 300 °C. A volume of 1 µL was injected in splitless mode. The total running time was 20 min.

**Figure 2:**
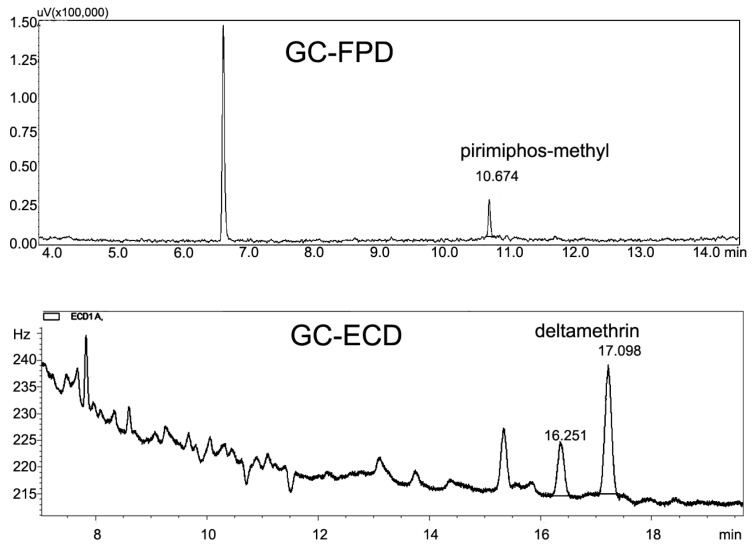
Typical GC chromatograms of pirimiphos-methyl (0.01 mg/kg) and deltamethrin (0.06 mg/kg) matrix-matched standard.

A Shimadzu GC-2000 instrument (Shimadzu, Kyoto, Japan) equipped with an AOC-20i auto-injector, an AOC-20s auto-sampler and a Flame Photometric Detector (FPD) was used for analysis of pirimiphos-methyl. A Shimadzu RTX-17 capillary column (30 m × 0.25 mm i.d. × 0.25 µm film thickness) was used for the GC separations, with nitrogen as carrier gas at a constant flow rate of 1.0 mL/min. The column temperature was initially at 120 °C (hold for 1 min), increased to 190 °C at the rate of 25 °C/min, and then to 230 °C at the rate of 10 °C/min, finally to 250 °C at the rate of 25 °C/min holding for 1.4 min. The temperatures of injector port and detector were all 250 °C and a volume of 1 µL was injected in splitless mode. The total running time was 12 min. Typical GC chromatograms of pirimiphos-methyl and deltamethrin are shown in Figure 2.

### 2.5. Statistical Method

The dissipation kinetics of pirimiphos-methyl and deltamethrin in rice were determined by plotting the residue concentration against time, and the maximum squares of the correlation coefficient obtained in this way were used to determine the equations of the best fit curves. For all the samples studied, exponential relationships were applied, corresponding to a first-order rate equation. Confirmation of the first order kinetics was further made graphically from the equation C_t_ = C_0_e^−kt^, where C_t_ represents the concentration of the pesticide residue at the time of t, C_0_ represents the initial deposits after application, and k is the degradation rate constant in per day. The half-life (t_1/2_) was calculated from the k value for each experiment, being t_1/2_ = ln(2)/k.

## 3. Results and Discussion

### 3.1. Method Validation

The modified QuEChERS procedure was evaluated for analysis of pirimiphos-methyl and deltamethrin residues in rice. A calibration curve of a matrix-matched standard calibration method was used in quantification of sample extracts during validation. The linearity of each pesticide was studied in the range of 0.01–1 mg/L with five calibration points (0.01, 0.06, 0.1, 0.5 and 1 mg/L for pirimiphos-methyl; 0.06, 0.1, 0.5, 1 and 2 mg/L for deltamethrin) in rice matrix. Linear calibration graphs were constructed by least-squares regression of concentration *vs.* peak area of calibration standards. Linearity values of pirimiphos-methyl and deltamethrin in rice matrix, calculated as determination coefficients (R^2^) were 1 and 0.9991, as shown in Table 1. Quantification was accomplished using the standard curve constructed by plotting analyte concentrations against peak area. To investigate the accuracy and precision of the method, recovery experiment contained five replicates spiked samples at three different levels (0.01, 0.1 and 0.5 mg/kg for pirimiphos-methyl; 0.06, 0.1 and 0.5 mg/kg for deltamethrin) were carried out.

**Table 1:** Recoveries, RSD, LOQ and regression equation of pirimiphos-methyl and deltamethrin in rice.

Pesticide	Spiked Level (mg/kg)	Average Recovery (n = 5%)	RSD (%)	LOQ (mg/kg)	Regression Equation	R^2^
Pirimiphos-methyl	0.01	96.3 ± 7.6	7.9	0.01	y = 6936300x + 26524	1
	0.1	107 ± 3.8	3.5			
	0.5	95.5 ± 1.9	2.0			
Deltamethrin	0.06	78.3 ± 3.3	3.8	0.06	y = 3207.4x + 66.442	0.9991
	0.1	106 ± 3.7	3.1			
	0.5	98.2 ± 3.2	2.9			

The average recoveries of pirimiphos-methyl and deltamethrin were 96.3%‒107.3 % and 78.3%‒106.2 %. Precision was studied as intraday precision (%, RSD), which was conducted by detecting five parallel spiked samples in one day at the mentioned three concentration levels, and they were ranged from 2.0% to 7.9%. The limits of quantification (LOQs) of pirimiphos-methyl and deltamethrin in rice were 0.01 mg/kg and 0.06 mg/kg.

### 3.2. Residue Dynamics of Pirimiphos-methyl and Deltamethrin

**Figure 3:**
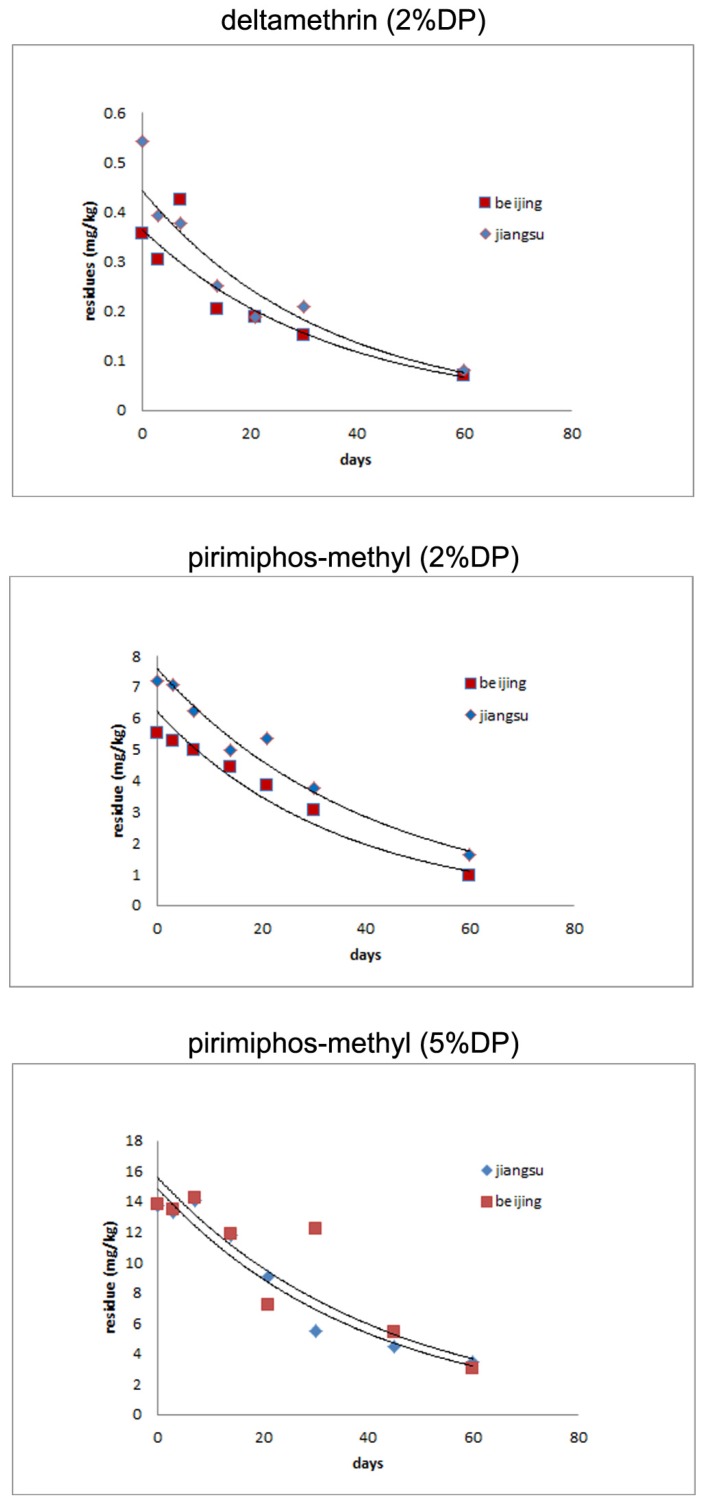
Results of dissipation dynamics of pirimiphos-methyl and deltamethrin in stored rice.

The results of dissipation dynamics of pirimiphos-methyl and deltamethrin in stored rice are shown in Figure 3. In the application of 2% pirimiphos-methyl and deltamethrin DP in stored rice, the initial deposits of pirimiphos-methyl were 7.2 mg/kg (Jiangsu, China) and 5.5 mg/kg (Beijing. China), and half-lives were 27.7 days (Jiangsu, China) and 23.9 days (Beijing, China). The initial deposits of deltamethrin were 0.54 mg/kg (Jiangsu, China) and 0.36 mg/kg (Beijing, China), and half-lives were 23.9 days (Jiangsu, China) and 24.8 days (Beijing, China). In the application of 5% pirimiphos-methyl DP in stored rice, the initial deposits of pirimiphos-methyl were both 13.8 mg/kg in Jiangsu and Beijing, and the half-lives were 26.7 days (Jiangsu, China) and 28.9 days in Beijing.

The dissipation dynamics from use of 2% DP in stored rice could be described by the following first-order kinetic equations: Pirimiphos-methyl: C = 7.5985e^−0.025t^ (Jiangsu, R² = 0.973), C = 6.2175e^−0.029t^ (Beijing, R² = 0.963); Deltamethrin: C = 0.4428e^−0.029t^ (Jiangsu, R² = 0.9379), C = 0.3641e^−0.028t^ (Beijing, R² = 0.9212). The dissipation dynamics of pirimiphos-methyl from the use of 5% DP application in stored rice could be described as follows: C = 14.854e^−0.026t^ (Jiangsu, R² = 0.9542), C = 15.58e^−0.024t^ (Beijing, R² = 0.8317).

### 3.3. Residues of Pirimiphos-methyl and Deltamethrin in Stored Rice

After the application of 2% DP (pirimiphos-methyl, 4.5–6.75 a.i. mg/kg; deltamethrin, 0.5–0.75 a.i. mg/kg) in stored rice, final residues of pirimiphos-methyl and deltamethrin were 1.6–4.5 mg/kg and 0.13–0.18 mg/kg (Table 2).

Final residues of pirimiphos-methyl from the use of 5% DP (pirimiphos-methyl, 10–15 a.i. mg/kg) in stored rice were 3.0–6.4 mg/kg (Table 3). The application rate of pirimiphos-methyl from 5% DP (10–15 a.i. mg/kg) was 2.2 times of that from 2% DP (4.5–6.75 a.i. mg/kg). Initial deposits of pirimiphos-methyl from the 5% DP application were also about 2.2 times of those from 2% DP application. Pirimiphos-methyl residues at 60 days PHI from 5% DP application were nearly twice those from 2% DP application, which was in step with its initial presence from two applications. A linear relationship was observed between the application rates and residues of pirimiphos-methyl after the application in stored rice. The CAC has established MRLs for pirimiphos-methyl (7 mg/kg) and deltamethrin (2 mg/kg) in rice. All final residues at 60 days PHI were lower than the MRLs. Therefore, these two pesticides are safe to be used in stored rice under recommended dosages.

**Table 2:** Final residues of 2 % pirimiphos-methyl and deltamethrin DP in stored rice.

Site	PHI (Days)	Pirimiphos-methyl		Deltamethrin	
		Application Rate a.i. mg/kg	Residue (mg/kg)	Application Rate a.i. mg/kg	Residue (mg/kg)
Beijing	30	4.5	3.1	0.5	0.17
	60		1.6		0.13
	30	6.75	4.0	0.75	0.13
	60		3.0		0.13
Jiangsu	30	4.5	3.6	0.5	0.15
	60		1.7		0.14
	30	6.75	4.5	0.75	0.18
	60		3.8		0.14

**Table 3:** Final residues of 5 % pirimiphos-methyl DP in stored rice.

Site	PHI (Days)	Pirimiphos-methyl	
		Application Rate (a.i. mg/kg)	Residue (mg/kg)
Beijing	45	10	4.5
	60		3.0
	45	15	5.4
	60		4.5
Jiangsu	45	10	4.5
	60		3.4
	45	15	6.4
	60		4.5

## 4. Conclusions

In this study, the dissipation and residues of pirimiphos-methyl and deltamethrin under storage condition were studied. A modified QuEChERS method with MWCNTs as r-DSPE material was applied in the analysis of pirimiphos-methyl and deltamethrin residues in stored rice after post-harvest treatments. The half-lives of pirimiphos-methyl and deltamethrin in rice under storage condition at two sites were in the range of 23.9–28.9 days. Residues from 5% pirimiphos-methyl DP application were significant higher than those from 2% pirimiphos-methyl and deltamethrin DP application. But none of the residues exceeded the MRLs established by the CAC. This study suggests that pirimiphos-methyl and deltamethrin could be used in stored rice when used at recommended dosages.
